# The biocontrol potential of endophyte *Bacillus velezensis* to reduce post-harvest tomato infection caused by *Rhizopus microsporus*

**DOI:** 10.1128/spectrum.01064-25

**Published:** 2025-10-27

**Authors:** Alicia Kock, Mmanoko Napo, Dionné Viviers, Oluwakemi V. Akinmoladun, Kazeem A. Alayande, Abdullahi Yusuf, Jessie Uehling, Teresa E. Pawlowska, Rasheed A. Adeleke

**Affiliations:** 1 Unit of Environmental Sciences and Management, North-West University 56405, Potchefstroom, South Africa; 2 Department of Zoology and Entomology, University of Pretoria 56410, Pretoria, South Africa; 3 Department of Botany and Plant Pathology, Oregon State University 2694, Corvallis, Oregon, USA; 4 School of Integrative Plant Science, Plant Pathology & Plant-Microbe Biology, Cornell University 5922, Ithaca, New York, USA; Pennsylvania State University, University Park, Pennsylvania, USA

**Keywords:** biocontrol, post-harvest, endophyte, *Bacillus velezensis*, secondary metabolites, *Rhizopus microsporus*

## Abstract

*Rhizopus microsporus* is a necrotrophic post-harvest pathogen that causes significant economic losses in the agricultural sector. To explore alternatives to conventional management strategies for the mitigation of post-harvest infections, we investigated the potential of two previously identified endophytic *Bacillus velezensis* strains as biological control agents. Through *in vitro* and *in vivo* experiments, we examined the mechanisms of biocontrol displayed by two *B. velezensis* strains (KV10 and KV15) against three *R. microsporus* strains (W2-50, W2-51, and W2-58). *In vitro* assays assessed co-cultivability and the inhibitory effects of *B. velezensis* against *R. microsporus*. The results demonstrated strain-specific antifungal activity with a reduction in fungal growth across treatments. Further analysis revealed that volatile organic compounds produced by *B. velezensis* contributed to its antifungal properties. To evaluate the biocontrol efficacy *in vivo*, tomato fruits were inoculated with *R. microsporus* and subsequently treated with *B. velezensis*. The results support the strain-specific reduction in tomato spoilage, yielding various spoilage rates observed across treatments. Our findings highlight the potential of *B. velezensis* as a promising biocontrol agent for the management of *R. microsporus* post-harvest infections in tomatoes. Further research is warranted to optimize the application *of B. velezensis* as a sustainable and environmentally friendly approach for controlling post-harvest diseases in tomatoes.

**IMPORTANCE:**

Our study shows the significance of improving sustainable agriculture by offering an alternative to the use of chemical fungicides in post-harvest applications. Opportunistic fungal pathogens like *Rhizopus microsporus* can have detrimental effects on post-harvest commodities like tomatoes. Post-harvest fungal infections are mainly controlled by chemical fungicides that pose health risks to humans and the environment. Utilizing biocontrol agents provides an environmentally safe alternative. Understanding the mechanisms of biocontrol employed by beneficial bacteria like *Bacillus velezensis* on fungal pathogens gives insight into safer, more environmentally friendly alternatives to protect food crops. Our results suggest that targeted microbial solutions can mitigate post-harvest losses.

## INTRODUCTION

Fungal infections contribute to about 20%–25% of post-harvest losses in fruits and vegetables annually (1). Among the fungi associated with post-harvest spoilage are species of the globally distributed genus *Rhizopus (*2–4) (order Mucorales, phylum Mucoromycota) (5). Mucoralean fungi are one of the most well-studied groups of early diverging fungi. Although generally saprotrophic and commonly found in soil and decaying organic material, they also act as opportunistic pathogens of plants and humans as well as post-harvest spoilage agents (6). Several species of *Rhizopus,* including *R. microsporus,* are considered model species with tremendous industrial and medical significance (5).

*Rhizopus microsporus* gained notoriety due to its association with the endosymbiotic bacteria *Mycetohabitans* spp (7). *R. microsporus* is the causative agent of rice seedling blight and sunflower head-rot disease (2, 8). Although *R. microsporus* pathogenicity in rice seedlings is attributable to secondary metabolites produced by endobacteria, the presence and role of endobacteria in *R. microsporus* causing sunflower head-rot disease remains unknown (9). The symbiosis between *R. microsporus* and *Mycetohabitans* is considered a model relationship in studying bacterial-fungal relationships (10). In this symbiosis, the endobacteria have been shown to completely control asexual reproduction and partially control sexual reproduction (10, 11). In addition to altering the reproduction of *R. microsporus, Mycetohabitans* spp. are responsible for host virulence in plants (8, 9). *Mycetohabitans* spp. are also considered a defensive symbiont of *R. microsporus,* where the bacteria-produced toxins fend off fungivorous amoeba and nematodes (12).

In addition to acting as field crop pathogens, *Rhizopus* spp. have also been associated with post-harvest losses in various vegetable commodities, including sweet potatoes, peaches, strawberries, and tomatoes, in which they behave as necrothrophs (1, 13). For instance, *Rhizopus stolonifer* is among the most common causes of post-harvest losses in fruit. Furthermore, *R. microsporus* has recently been identified as being highly abundant in post-harvest spoiled tomatoes (14). Tomato fruits are especially prone to post-harvest fungal infection during transport and handling, where sustained damages create an infection point for necrotrophic fungi like *Rhizopus* spp. (15). Furthermore, tomatoes have a very short post-harvest shelf-life due to their high ethylene production that causes rapid ripening of the fruit (16). These vulnerabilities result in significant losses of tomatoes annually and raise the need to control and mitigate post-harvest infections and decrease economic loss (17, 18).

Chemical pesticides are widely used in agriculture, and this is no different in tomato production. However, these pesticides have harmful effects on the environment by degrading soil quality to the point of making it unusable for future agricultural practices (18). They pose a further risk to human and animal health by remaining on the crops after harvest (18). Moreover, pesticide levels are beyond the control of the consumer (19). Finally, routine use of fungicides to mitigate fungal infections increases the risk of fungicide resistance (20–22). To reduce the risks associated with chemical pesticides, there has been a rapid development in biotechnology and the application of biological control agents in the processing of post-harvest fruits and vegetables. Among microorganisms that can be applied as biocontrol agents are endophytic bacteria that reside asymptomatically in plant tissues (18, 23).

Endophytes are defined as either fungi or bacteria asymptomatically residing in plant tissue (23). The ability of endophytes to produce metabolites and regulate their host’s physiology has led to increased interest from researchers worldwide (23). Endophytes have the potential to promote growth in host plants and are often referred to as plant growth-promoting endophytes (23, 24). Some of the most well-studied species of plant-growth-promoting endophytes belong to the genus *Bacillus* (23, 24). Several *Bacillus* species have been shown to improve plant growth by directly affecting the plant’s innate immune response system or indirectly by inhibiting plant pathogens through the secretion of antimicrobial compounds (18, 23, 24).

Recently, *Bacillus velezensis* has gained research interest as a rapidly growing potential biological control agent (23, 24). *Bacillus* spp. display numerous mechanisms of biocontrol, including through stimulation of induced systemic resistance in plants, competition with pathogenic microbes, and the production of various secondary metabolites (25). *Bacillus* spp. are considered excellent root colonizers, and when associated with plants, they can facilitate induced systemic resistance in their host (25). The synthesis of secondary metabolites is another mechanism through which *Bacillus* spp. protect their plant hosts from pathogens. Several antimicrobial peptides (AMPs), including subtilisin, amylocyclicin, and subtilin, are produced by *B. velezensis* and act as antimicrobial agents against gram-positive bacteria (25). Lipopeptides act as anti-fungal agents, with a general mechanism of penetrating and disrupting the membrane permeability of the target pathogens (25). Polyketides are another group of secondary metabolites produced by *B. velezensis* capable of downregulating genes related to pathogen virulence, cell division, and cell wall synthesis (25). *Bacillus* spp. further produces volatile organic compounds (VOCs) that inhibit the growth of the target pathogens at a distance (25) and underpin the biocontrol capability of *B. velezensis* through disrupting fungal cell membrane permeability (26–29). Although *B. velezensis* is a known endophyte capable of inducing systemic resistance in plants, this study is focused on its non-endophytic biocontrol potential when applied post-harvest.

We aimed to explore the potential biological control capabilities of *B. velezensis* against the necrotrophic fungus *R. microsporus,* which, depending on its genotype, harbors the endosymbiotic bacteria *Mycetohabitans* spp or is endosymbiont-free. Both organisms were sourced from nature and applied to commercially grown tomato fruits. The specific objectives of the study were to: (i) investigate the *in vitro* antagonistic effects of *B. velezensis* on *R. microsporus,* (ii) analyze the biocontrol impact of VOCs produced by *B. velezensis* and their *in vitro* effect against *R. microsporus,* and (iii) investigate the *in vivo* application of *B. velezensis* against *R. microsporus* using tomato fruit as a model.

## MATERIALS AND METHODS

### Isolation and identification of fungal isolates

#### Isolation and identification of *Rhizopus* sp.

The fungal strains used in this study were isolated and identified by following a modified version of the methods described by Benny (30). Briefly, a small amount of rhizosphere soil was sprinkled onto a thin layer of wheat germ agar (WgA) prepared according to the methods described in Benny (30). No antibiotics were added to the medium. The inoculated WgA petri dishes were incubated for 5–7 days at room temperature (25°C), followed by repetitive subculturing to obtain pure cultures of the fungus. The fungal genomic DNA (gDNA) was extracted using the Quick DNA Fungal/Bacterial Miniprep Kit (Zymo Research) with a few modifications: mycelia were vortexed for 30 min, and only 70 µL elution buffer was added. Using a NanoDrop 1000 spectrophotometer (ThermoFisher Scientific, US), the quality and quantity of DNA were confirmed. The fungal 28S large subunit (LSU) rRNA gene was amplified using the LROR (5’- ACCCGCTGAACTTAAGC-3′) and LR7 (5’- TACTACCACCAAGATCT-3′) forward and reverse primers (Integrated DNA Technologies, SA), respectively (31, 32), using the following PCR conditions: initial denaturation at 95°C for 1 min, 30 cycles 95°C for 30 s, 58°C for 45 s and 72°C for 45 s, a final extension of 7 min at 72°C. Successful amplification was confirmed using 1.5% (vol/vol) agarose TAE-electrophoresis of PCR amplicons visualized using the GelDoc Imaging System, BioRad. PCR amplicons were submitted for Sanger Sequencing at Inqaba BiotecTM, SA, using the same 28S LSU primers as mentioned above. The obtained sequences were assembled and edited using Geneious Prime v.2.1 and exported to Mesquite v.3.81 (33) where sequences were aligned with the MUSCLE plug-in (34). Best-fit nucleotide substitution models and maximum likelihood phylogenetic reconstructions were carried out in RAxML-GUI v.2.0 (35). Phylogenetic trees were inferred with the GTR + Gamma-distributed rate variation model and 1,000 bootstrap replicates. The obtained trees were edited using iTOL (36). From there, isolates identified as *R. microsporus* W2-50, W2-51, and W2-58 were selected for the following experiments. Isolates were stored at −80°C in glycerol until further experiments were conducted.

#### Molecular identification of bacterial endosymbionts of *Rhizopus*

Because bacterial endosymbionts play an important role in *Rhizopus* pathogenicity in plants (37), the fungal isolates were molecularly screened for the presence of potential endobacteria. The extracted gDNA samples were subjected to PCR amplification using 16S gene rRNA 27F (5′-AGAGTTTGATCCTGGCTCAG-3′) and 1492R (5′-GGTTACCTTGTTACGACTT-3′) forward and reverse primers (Inqaba BiotecTM, SA), respectively (11, 38, 39). The following PCR conditions were used: initial denaturation at 95°C for 1 min, 30 cycles at 95°C for 30 s, 61°C for 30 s, and 68°C for 30 s, a final extension of 7 min at 68°C. Further processing and analysis were conducted as above.

### Isolation and identification of plant endophytic bacteria

The bacterial endophytes were isolated from *Elytropappus rhinocerotis* leaves and identified as *B. velezensis* as described in Alayande *et al*. (40).

### Strains and culture conditions

*Rhizopus microsporus* strains were revived from −80°C glycerol stocks and maintained on malt yeast extract agar (MYA). The isolates were incubated at 28°C for 7–10 days before being used in the *in vitro* and *in vivo* assays. Isolates of *B. velezensis* were maintained on nutrient agar consisting of 3 g yeast powder (Sigma-Aldrich), 5 g peptone powder (Sigma-Aldrich), 5 g sodium chloride, and 15 g bacteriological agar (Millipore) dissolved in 1 L of distilled water. The isolates were incubated at 37°C for 24 h and kept at 4°C before being used in the respective assays.

Two *B. velezensis* strains, KV10 and KV15, were selected for this study based on their previously revealed genome properties (41), providing a strong foundation for their hypothesized role as biocontrol agents. Specifically, genomic sequences of KV10 and KV15 revealed the capacity for biosynthesis of eight and six distinct antimicrobial metabolites, respectively. Their genetic potential supports their use in experiments aimed at mitigating surface-level post-harvest fungal infections. The three *R. microsporus* strains were selected due to their association with *Mycetohabitans,* which is potentially responsible for the virulence of *R. microsporus*.

### *In vitro* determination of antagonistic effects of *B. velezensis* on *R. microsporus*

#### Agar-based co-culture

**Fig 1:**
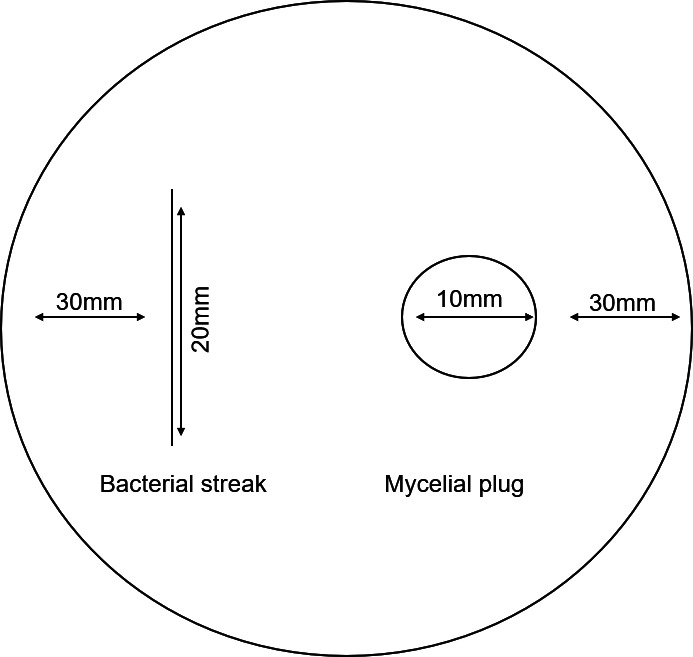
The experimental design for co-culturing of fungi and bacteria showing the bacterial streak on the left and the mycelial plug on the right.

Co-culture assays were performed on potato dextrose agar[1]PDA (Sisco Research Laboratories), using a streak-cultured bacterial colony and a 10 mm mycelial plug (42). The mycelial plug of *R. microsporus* was placed upside-down on a PDA plate 30 mm from the side of the plate, after which a 20 mm bacterial streak was placed 30 mm from the other side of the agar plate (Fig. 1). The plates were incubated at 28°C for 3 days, and the fungal colony was measured daily.

The inhibition rate was calculated using the following formula (22)


$$Inhibition\;rate\;(\%)=\frac{(Average\;of\;control\;group\;colony\;diameter-average\;of\;treatment\;group\;colony\;diameter)}{Average\;of\;control\;group\;colony\;diameter}\times 100$$

### Volatile organic compounds analysis

#### Collection and analysis of headspace VOCs

Headspace volatiles produced by bacteria were collected following the method described below. Briefly, the nutrient broth medium was poured into 20 mL headspace vials, closed with parafilm, and sterilized. Each bacterial culture was inoculated into the sterilized nutrient broth in the headspace vials and incubated in a shaker at 28°C at 150 rpm for 3 days for the bacteria to grow. After a 3-day incubation period, volatiles were collected by inserting an SPME fiber (50/30 μm DVB/CAR/PDMS, Stableflex 24Ga, Merck KGaA, Damstadt, Germany) into the headspace of the vial containing the bacterial isolates for 12 h at room temperature (24°C). The SPME fiber was then desorbed into the injection port of a Shimadzu QP 2000 SE (Shimadzu Incorporation, Japan) Gas Chromatography- Mass spectrometer (GC-MS) set at 2500°C for 3 min on an Rtx-5MS Restek column (Restek Corporation, Bellefonte, PA, USA). Helium was used as the carrier gas at a flow rate of 1 mL/min with the oven temperature initially programmed at 300°C for 1 min, increased at 50°C per minute to 1,500°C, then at 150°C per minute to 2,000°C, and increased at 300°C to a final temperature of 3,000°C, which was held for another minute. The transfer line and ion source temperatures of the MS were set at 2,800°C and 2,500°C, respectively, and mass spectra were obtained in the electron ionization mode (EI) at 70 eV with a mass range of 35–600 Da, scanning at 2,000 in 0.30 s intervals. Volatiles were tentatively identified by comparing their mass spectra with those from commercial libraries NIST 09 and Wiley 09. All analyses were performed in triplicate.

### *In vitro* determination of antagonistic effects of *B. velezensis* VOCs on *R. microsporus*

**Fig 2:**
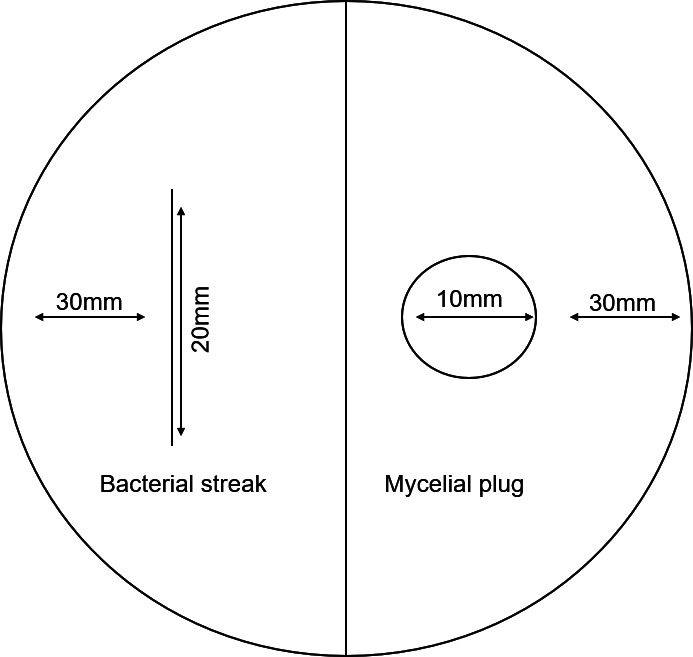
The experimental design for exposing *R. microsporus* strain to VOCs produced by *B. velezensis* in a split-compartment Petri dish.

To test the effects of VOCs produced by *B. velezensis* strains KV10 and KV15 on the mycelial growth of three *R. microsporus* strains, a split-compartment Petri-dish assay was performed following a protocol adapted from (20). Specifically, the one compartment of the split-compartment plates contained Luria Bertani (LB) media, and the second compartment contained PDA. The compartment containing the PDA was inoculated with a 10 mm mycelial plug placed upside-down on the surface of the PDA media. The compartment containing the LB media was streak-inoculated with a 20 mm streak of bacteria (Fig. 2). Each bacterial strain was tested against each fungal strain in five replicates. Control plates contained only fungal inoculations on the PDA side. The plates were incubated for 3 days at 25 ±2 °C. The fungal colonies were measured daily, and the inhibition rate was calculated with the following formula (22):


$$Inhibition\;rate\;(\%)=\frac{\left(\text{Average of control group colony diameter}-\text{average of treatment group colony diameter}\right)}{\text{Average of control group colony diameter}}\times 100$$

### *In vivo* test of *B. velezensis* as a potential biological control agent

#### Preparation of tomato fruit

Fresh, healthy tomato fruits (*Solanum lycopersicum* – Heinz 1370) of roughly the same size were purchased from a local supermarket. The tomatoes were surface sterilized by first washing them thoroughly under running water, then soaking them in freshly prepared 70% ethanol for 5 min, and soaking them in 2% sodium hypochlorite for 15 min before finally rinsing them in autoclaved distilled water. The tomato fruits were left to air dry in a sterile laminar flow cabinet.

#### Preparation of bacterial and fungal inoculum

Bacterial isolates were grown in nutrient broth for 24 h at 37°C. The bacterial cells were washed three times with phosphate-buffered saline (PBS) (41), and the optical density was adjusted to 0.2 at a wavelength of 600 nm (43).

A spore suspension was prepared by flooding an actively growing plate of *R. microsporus* with 500 µL of PBS. The flooded plate was then lightly scraped to release the spores, and the suspension was collected in a sterile Falcon tube. The spores were then suspended in autoclaved distilled water, and the optical density at 420 nm was adjusted to 0.2 (15).

#### Inoculation of tomato fruits

**Fig 3:**
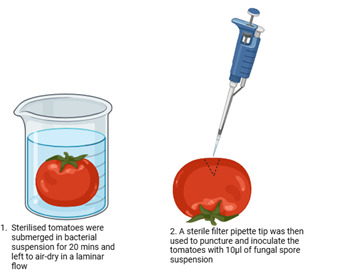
Experimental design for the puncture inoculation treatment to test the efficacy of *B. velezensis* as a potential biocontrol agent against *R. microsporus*.

The inoculation of tomato fruits was performed in two separate rounds of treatment following two separate methods for optimal determination of biocontrol efficacy against *R. microsporus*. For the first treatment, puncture inoculation (Fig. 3), the sterilized tomatoes were left to soak in the bacterial suspension for 20 min, and the tomatoes were then left to air-dry in a laminar flow (21). A sterile filter pipette tip was then used to puncture and inoculate the tomatoes with 10 µL of spore suspension. Careful consideration was taken to ensure the created wounds were equal in depth.

**Fig 4:**
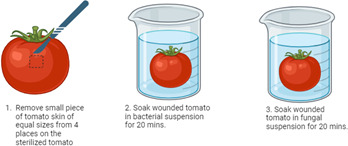
Experimental set-up for the exocarp peel inoculation treatment to test the efficacy of *B. velezensis* as a potential biocontrol agent against *R. microsporus*.

For the second treatment, exocarp peel inoculation (Fig. 4), three incisions were created across the horizontal profile of the tomato with a sterile scalpel. This was carefully done to only remove the exocarp of the tomato and ensure that the created wounds were equal in size. The tomatoes were submerged in the bacterial suspension as described above and left to air-dry in a laminar flow. For the fungal inoculation, the air-dried tomatoes were then submerged in the fungal spore suspension for 20 min.

The treatments consisted of fungi-bacteria combinations and were conducted in five replicates for each treatment. Controls were set up for bacteria-only and fungi-only treatments as well as untreated replicates. Tomato fruits were sealed in sterile plastic bags and incubated at 25°C ±2 °C for 7 days (15). Disease symptoms in the fruit were noted daily. Upon termination of the experiments, a disease incidence was calculated as a percentage adapted from (14).


$$Disease\;incidence\;\left(\%\right)=\;\frac{number\;of\;infected\;fruit\;}{total\;number\;of\;fruit\;per\;treatment}\times 100$$

### Koch’s postulate of pathogenicity

To fulfill Koch’s postulate of pathogenicity, the spoiled tomatoes were used to re-isolate the disease-causing fungal isolates. After the completion of the puncture inoculation and exocarp peel inoculation experiments, sterile swabs were used to swab the surface of the tomato, focusing on the inoculated wounds. The swabs were plated on PDA agar and sub-cultured until pure colonies were obtained. Genomic DNA of each isolate was obtained using the Quick DNA Fungal/Bacterial Miniprep Kit (Zymo Research). The gDNA samples were subjected to both 28S and 16S rRNA gene PCR amplification, respectively, following the same conditions as previously described. This was done to confirm the presence of the fungal pathogen as well as to screen for the potential presence of the endosymbiont *Mycetohabitans* spp. Once again, the successful amplifications were confirmed on a 1.5% (vol/vol) Agarose TAE-gel electrophoresis, and the amplicons were sent for both 16S rRNA SSU gene and 28S LSU gene Sanger sequencing at Inqaba (Inqaba BiotecTM, SA). Phylogenetic inferences were also achieved following the protocol as previously described.

### Statistical analyses and visualizations

Statistical analyses and visualizations for the data obtained were performed using R statistical software (version 4.5.1) within the RStudio integrated development environment. For the visualization of the relative abundance of VOCs produced by different bacterial isolates, a heatmap was generated using the ggplot2 package in R. The data, which consisted of GC-MS peak areas for various VOCs and isolates, was first reshaped into a long format using the tidyr::gather() function. A heatmap was then created, with isolates on the x-axis, VOCs on the y-axis, and peak areas represented by a color gradient. The scale_fill_gradient() function was used to set the color scale, and the theme_minimal() function was applied for a clean and minimalist aesthetic.

Data obtained from *in vitro* co-culturing and VOC experiments were imported as comma-separated values (csv) files. Descriptive statistics for the inhibition rate were calculated for each unique combination of treatment and time point. This involved computing the mean and standard deviation for the inhibition rates using functions from the dplyr and rstatix packages. Prior to conducting hypothesis tests, the assumption of normality for the inhibition rate within each treatment and time point was assessed using the Shapiro-Wilk test. This was achieved by using the Shapiro_test() function for the rstatix package. For the co-culturing experiment, the data were normally distributed, and the significance level α = 0.05 was used to interpret the test results. A one-way analysis of variance (ANOVA) was performed to evaluate the main effects of treatment and time_point, as well as their interaction on inhibition rate. The aov() function in base R was used. Following the ANOVA, Tukey’s Honestly Significant Difference (HSD) post-hoc test was conducted to identify specific pairwise differences among the Treatment:Time_point interaction groups that were statistically significant. A significance level of α = 0.05 was applied for all hypothesis tests. Subsequently, the data for the split-compartment experiment were non-normally distributed. The Kruskal-Wallis rank sum test, a non-parametric approach, was therefore used to analyze this data set. Two separate tests were conducted to determine the effects of treatment and the effects of time. To visualize the results, a bar graph was generated using ggplot2. The plot displayed the mean inhibition rate for each group, with error bars representing the standard error. A letter-based ranking system, derived from the Tukey HSD post-hoc test, was used to indicate significant differences between groups. Groups sharing the same letter are not statistically different at a significance level of *P* > 0.05.

The incidence of tomato spoilage during the *in vivo* experiments was analyzed by comparing the fungal-only controls to the fungal vs bacteria treatments. To determine the statistical significance of the bacterial treatments, a series of pairwise comparisons was conducted using Fisher’s exact tests. For each comparison, a 2 × 2 contingency table was constructed, detailing the number of spoiled and not spoiled tomatoes for each group. The base stats package was used, and a *P*-value < 0.05 was statistically significant. Although uninoculated control replicates and bacteria-only control replicates were included in the experiment, these tomatoes were removed from statistical analysis since the aim is to determine the effect of the biocontrol agent on the fungi *in vivo*.

## RESULTS

### Identification of *R. microsporus* isolates and bacterial endosymbionts

**Fig 5:**
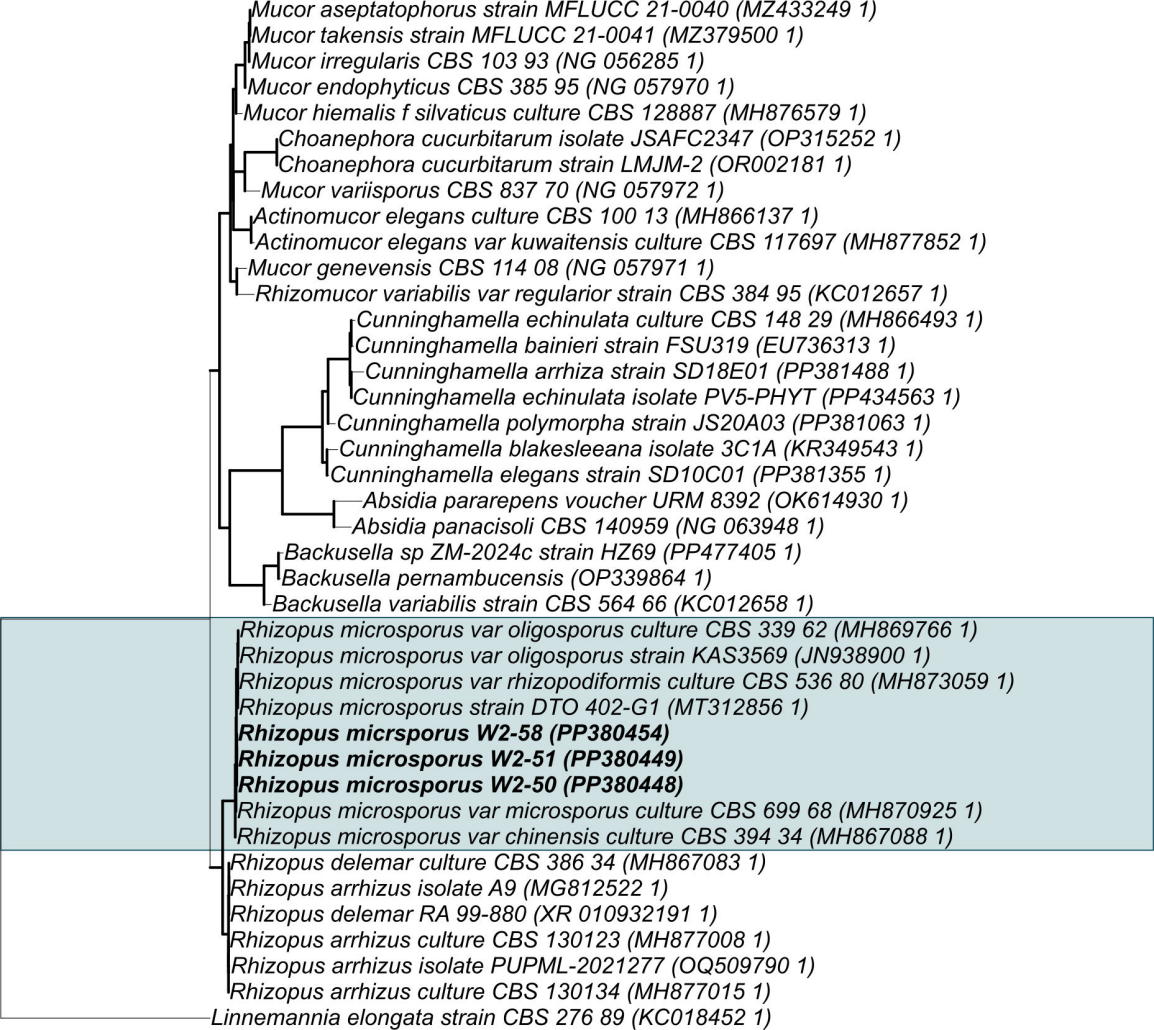
Phylogenetic tree based on the 28S large subunit rRNA gene sequences from selected *R. microsporus* isolates constructed using RAxML with the GTR + Gamma substitution model and 1,000 bootstraps. Bold branches represent bootstrap values > 70%. *Linnemannia elongata* strain CBS 276 89 was selected as the outgroup for the phylogenetic inference.

Three fungal isolates obtained from South African desert soil samples were selected for the experimental setup. Phylogenetic reconstruction of the 28S LSU rRNA gene sequences indicated that these strains belonged to *R. microsporus* (Fig. 5). Further molecular screening of these three *R. microsporus* strains revealed the presence of potential endobacteria *Mycetohabitans* sp. ( Fig. S3) previously implicated in *R. microsporus* virulence to plant hosts(36). The ability of these *R. microsporus* isolates to cause post-harvest spoilage and their susceptibility to inhibition by a common biocontrol agent were examined in the course of the study.

### *In vitro* determination of antagonistic effects of *B. velezensis* against *R. microsporus*

#### Inhibition rate (%)

**Fig 6:**
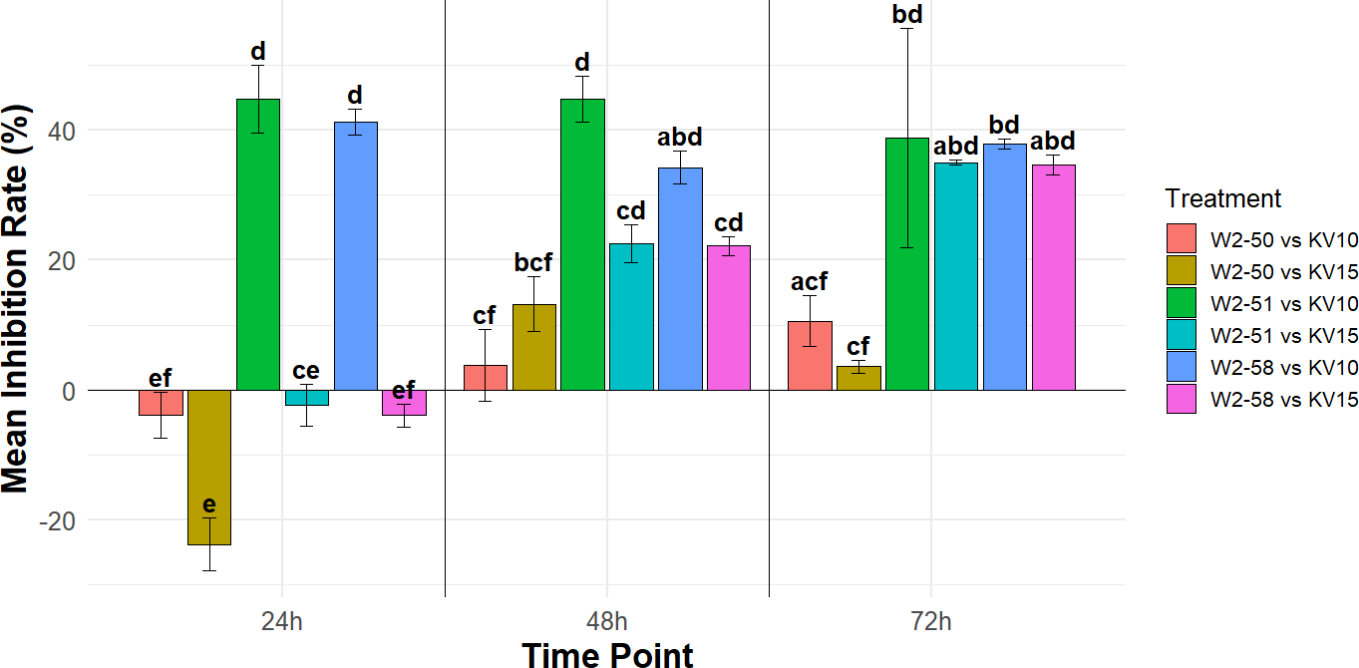
Inhibitory rate of three *R. microsporus* strains co-cultured with two *B. velezensis* strains calculated at 24-, 48-, and 72-h incubation periods. Positive values indicate successful inhibition, standard error of the while negative values indicate unsuccessful inhibition of fungal strains. Error bars represent the standard error of the mean (*n* = 5). Bars that do not share a common letter are statistically different from one another according to Tukey’s HDS test (*P* < 0.05).

The *R. microsporus* colony diameter in co-culture with *B. velezensis* was measured over a 72-h period (Fig. S1). The average between five replicates for each treatment was calculated and used to determine the inhibition rate of each treatment. Figure 6 shows the inhibition rate over an incubation period of 3 days, with positive values indicating a successful inhibition and negative values indicating unsuccessful inhibition, in which case the growth of the fungus was enhanced. Although the patterns varied, observed inhibition rates indicated that *B. velezensis* KV10 was more successful at inhibiting the growth of *R. microsporus* W2-51 and W2-58 as opposed to W2-50. Interestingly, *B. velezensis* KV15 initially augmented the growth of all three fungal isolates but started to inhibit their growth after 48 h.

### *In vitro* determination of antagonistic effects of *B. velezensis* VOCs on *R. microsporus*

#### VOC analysis results

**Fig 7:**
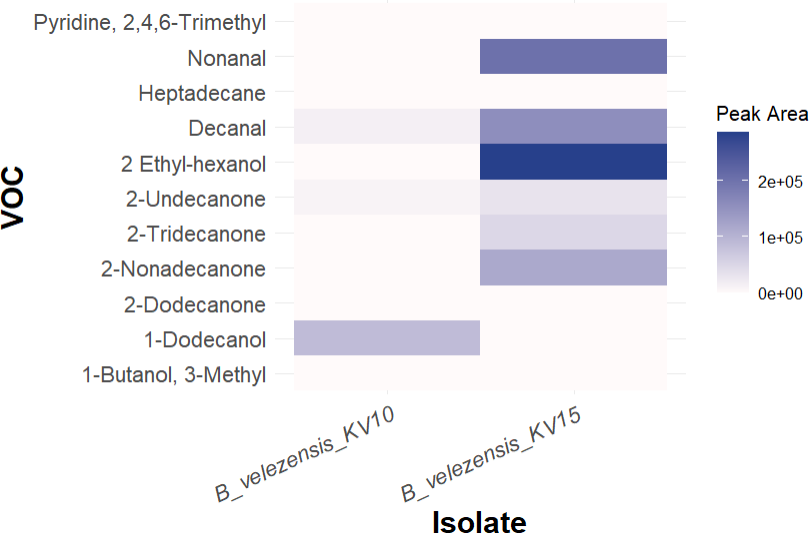
Relative abundance of volatile organic compounds (VOCs) produced by *B. velezensis* KV10 and *B. velezensis* KV15. Each cell on the heatmap represents the peak area of the specific VOC produced by each bacterial isolate. The darker shades indicate higher VOC production, whereas the lighter shades indicate lower VOC production.

The VOCs produced by both *B. velezensis* KV10 and *B. velezensis* KV15 were analyzed with gas chromatography-mass spectrometry. These analyses revealed that more VOCs were produced by *B. velezensis* KV15 than by *B. velezensis* KV10 (Fig. 7). The identified VOCs included two ethyl-hexanols, undecanone, 1-dodecanol, and 2-tridecanone, which are common antifungal VOCs produced by a variety of bacterial isolates (25, 44, 45).

#### Inhibition rate (%) after exposure to VOCs

**Fig 8:**
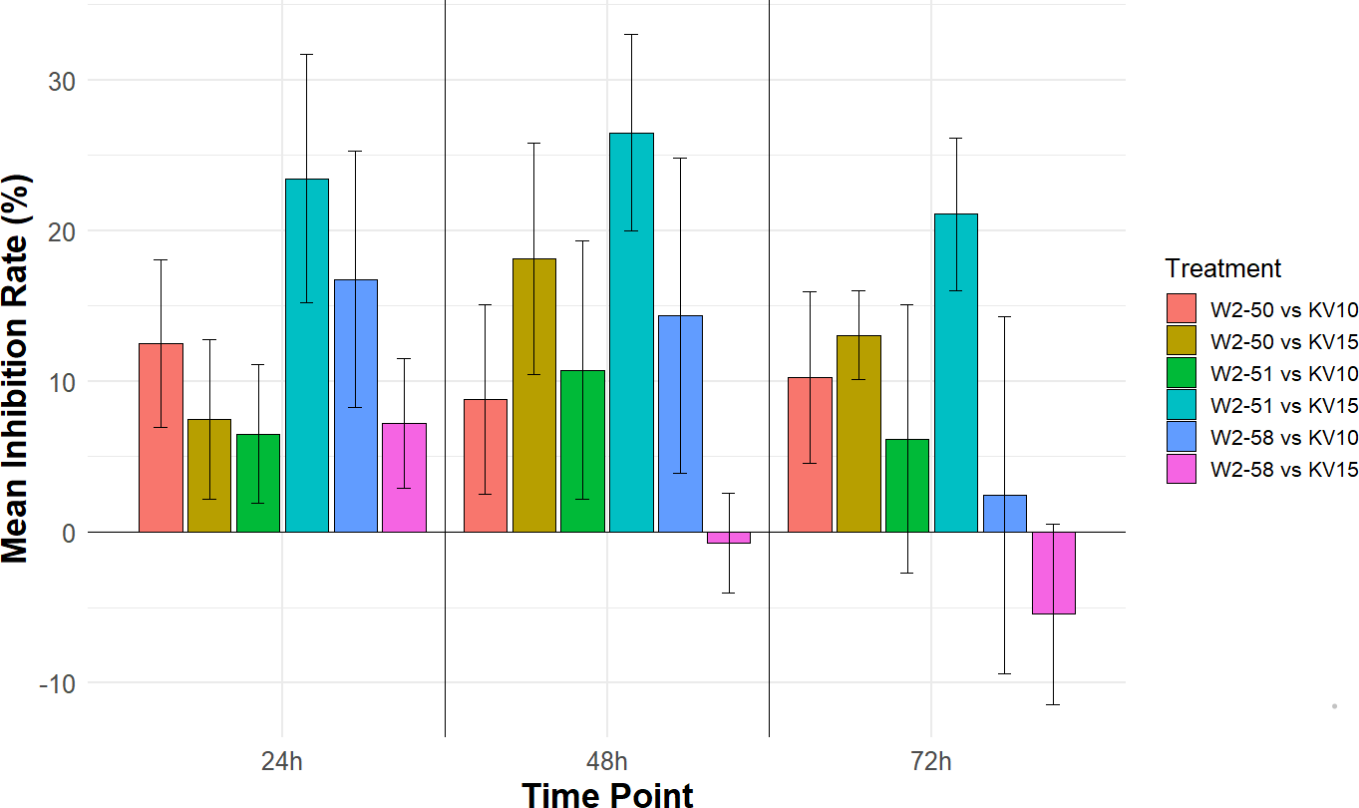
Inhibitory rate of three *R. microsporus* strains cultured against two *B. velezensis* strains in a split compartment agar plate calculated at 24-, 48-, and 72-h incubation periods. Positive values indicate successful inhibition, while negative values indicate unsuccessful inhibition of fungal strains. Data are represented as the mean ± standard error of the mean (*n* = 5). A Kruskal-Wallis test revealed no statistically significant differences among treatment groups at any time points (*P* > 0.05).

To examine the effects of VOCs produced by *B. velezensis* on *R. microsporus* growth, bacteria and fungi were grown in split-compartment agar plates (Fig. S2). The fungal colony diameters were measured over a 72 h period and used to determine the inhibition rate in each treatment. Various degrees of inhibition could be observed across treatments (Fig. 8). Interestingly, *B. velezensis* KV15 augmented the growth of *R. microsporus* W2-58 rather than inhibiting it at the 48 h time point. However, the Kruskal-Wallis hypothesis test revealed no statistical difference between treatment groups at any time point.

### *In vivo* test of *B. velezensis* as a potential biological control agent

**Fig 9:**
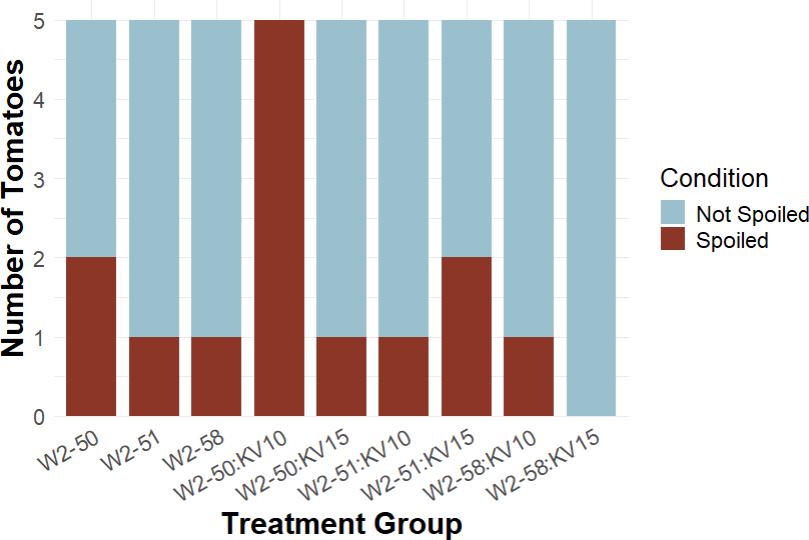
Spoilage indices observed in tomato fruits treated with *B. velezensis* and inoculated with *R. microsporus*. Pairwise comparisons with Fisher’s exact test revealed no significant difference in treatments.

Figure 9 summarizes the spoilage observed during the *in vivo* testing of *B. velezensis* strains KV10 and KV15 against three *R. microsporus* strains introduced by puncture inoculation (Fig. 3). In the case where spoilage was not induced with the *R. microsporus* inoculation, naturally occurring spoilage could be observed in these treatments. However, since the spoilage was not due to the experimental inoculation, these tomatoes have been removed completely from further statistical analysis. Complete spoilage of the tomatoes inoculated with *R. microsporus* W2-50 and KV10 was observed, whereas only one out of five tomatoes spoiled when inoculated with *R. microsporus* W2-50 and KV15. However, statistical analysis showed no significant differences (*P*-value > 0.05) between the number of tomatoes inoculated with *R. microsporus* only versus inoculated with *R. microsporus* and *B. velezensis*. The Fisher’s exact test also revealed no statistical significance between any of the treatments. However, given the outcome of our *in vitro* experiments, it is possible that an increase in the number of replicates per treatment could result in statistically significant results. Following the principle of Koch’s postulate for pathogenicity testing, we were able to re-isolate the *R. microsporus* strains from tomatoes inoculated with fungi and confirm their association with *Mycetohabitans* through molecular screening (Fig. S3).

**Fig 10:**
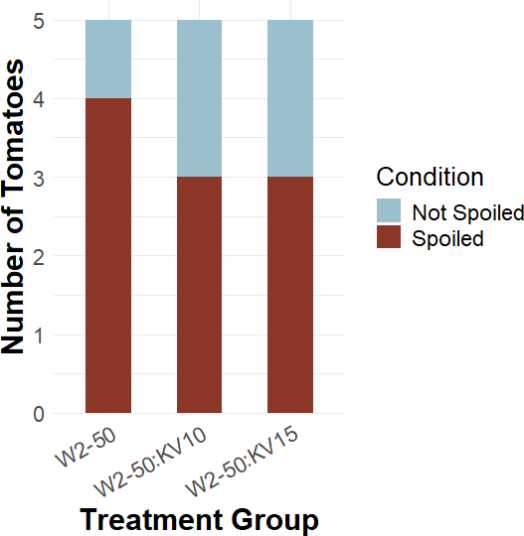
Spoilage indices observed in tomato fruits treated with two *B. velezensis* strains, KV10 and KV15, and inoculated with *R. microsporus* strain W2-50. Pairwise comparisons with Fisher’s exact test revealed no significant difference between treatment groups.

Following the results from the puncture inoculation treatment (Fig. 9), we decided to repeat the experiment with an alternative method, the exocarp peel inoculation (Fig. 4). However, as a result of *R. microsporus* isolate W2-50 inoculated with KV10, showing spoilage regardless of the biocontrol treatment, we decided to only inoculate with *R. microsporus* isolate W2-50. Figure 10 depicts the number of spoiled vs non-spoiled tomatoes per treatment. Once again, it is important to note that uninoculated controls and bacteria-only results were removed from further statistical analysis. Another important observation is that the fungal growth occurring on the treated tomatoes was not in the inoculation points we created. Instead, the fungi established themselves on the stem scar of the tomato, and no apparent spoilage was observed (Fig. S4). However, the pairwise comparisons with Fisher’s exact test revealed no statistical significance (*P*-value > 0.05) across treatment groups. Following the principle of Koch’s postulate for pathogenicity testing, we re-isolated the *R. microsporus* strains associated with *Mycetohabitans* sp (Fig. S3).

## DISCUSSION

This study explored the application of a potential biocontrol bacterial endophyte to tomato fruits to mitigate post-harvest infections by a fungal pathogen. The results revealed the possibility of using *B. velezensis* as a mitigating strategy for post-harvest fungal infections caused by *R. microsporus*.

### Biocontrol capabilities of two *B. velezensis* strains against three *R. microsporus* strains: a co-culture approach

A previous study by Alayande *et al.* (42) analyzed the secondary metabolite gene clusters in *B. velezensis* KV10 and KV15 and predicted that both strains are able to produce the non-ribosomal peptide fengycin, a lipopeptide known to disrupt the phospholipid bilayer of filamentous fungi, including *R. stolonifer,* compromising their cell membrane (46, 47). Indeed, growing these fungal and bacterial isolates in co-culture resulted in inhibition of *R. microsporus* strains. However, further analysis is needed to confirm the role of fengycin in this phenomenon. Inhibition of *R. microsporus* by *B. velezensis* was strain-specific, a pattern consistent with previous reports on the effects of *B. velezensis* on *Fusarium* spp. and *R. stolonifer* (48, 49).

### Volatile organic compounds (VOCs) produced by *B. velezensis* with antifungal capabilities

Analysis of the VOCs produced by *B. velezensis* KV10 and KV15 revealed compounds previously reported to have antifungal activity (45, 50–52). For example, two ethyl-hexanols produced by *B. velezensis* KV15 were found to be effective against the *Fusarium* spp. (53). Importantly, it was also identified as an indoor air pollutant and one of the leading causes of sick building syndrome when inhaled by humans (54). As this compound might pose a risk to human health, it is imperative to further investigate the presence of 2-ethylhexanol in potential biocontrol agents. Similarly, 2-undecanone, 1-dodecanol, and 2-tridecanone have been identified previously in *Bacillus* spp. and reported to show fungicidal efficacy against *Botrytis cinerea, Monilinia* spp., *Alternaria solani*, *Verticillium dahliae,* and *Fusarium oxysporum*, respectively (50–52, 55). Likewise, 2-nonadecanone exhibited anti-fungal activity against *Penicillium* spp. (56). Another study (57) highlighted that interactions between fungi and bacteria vary and include competition and synergistic interaction. They further found that VOCs produced in co-culture have a higher inhibition efficacy against phytopathogens (57).

*Bacillus* spp. display different antifungal strategies (25, 58), and this is highlighted in the *in vitro* experiments, where different rates of inhibition were observed between directly co-culturing *B. velezensis* with *R. microsporus* as opposed to exposing *R. microsporus* to *B. velezensis* VOCs*.* Although the exact mechanism of biocontrol is not confirmed, it is evident that the secondary metabolites produced by *B. velezensis* have an effect on *R. microsporus* strains. However, the potential mechanisms at play could include inhibition of pathogenicity-related genes, downregulation of energy metabolism-related genes, or cell-structure degradation (44). Although we show *in vitro* evidence of the effects of VOCs produced by *B. velezensis* against *R. microsporus,* future studies should aim to validate these effects through pure compound assays. Following this approach might give insight into the specific volatile compounds at play during fungal inhibition. A study fumigating apples infected with *Botryosphaeria dothidea* with *B. velezensis* VOCs further emphasizes the need for more studies investigating the practical application of VOCs in conjunction but also as pure compounds (59). Aside from VOC production by potential biological control agents, studies have investigated the role of biomass production, secondary metabolite production, competition for nutrients and space, as well as direct cellular interaction between the biocontrol agent and the fungus (15, 60). These studies suggest a synergistic role between biocontrol mechanisms; however, considering the broad range of biocontrol agents tested against *Rhizopus* spp., there might also be synergies between biocontrol agents worth investigating.

Both *in vitro* studies showed varying degrees of inhibition, but we also observed isolated cases where *B. velezensis* was unable to inhibit the growth of *R. microsporus.* This could potentially be attributed to the hormesis effect (61, 62). Hormetic effects have been reported where certain fungicides were able to augment fungal growth at low concentrations (56). However, it has also been reported that *Beauveria bassiana,* a potential fungal biopesticide, induced hermetic effects in *Myzus persicae* (63). The potential induced hormetic effects are very important to consider when evaluating biological control potential as well as establishing the effective concentration to administer.

### *In vivo* test of *B. velezensis* as potential biological control agent

During the puncture inoculation, all three *R. microsporus* strains were used and treated with both *B. velezensis* strains. This resulted in various spoilage incidents across treatments without conclusive results and led to the exocarp peel inoculation. Evidently, biocontrol capabilities were strain-specific across treatments. The exocarp peel inoculation further resulted in no apparent spoilage, and fungal growth was only observed in the tomato stem scar. These results suggest that the application of a biocontrol agent might lower the infection severity and redirect the infection. Due to the variability in results, we suggest that future studies focus on increasing the number of replicates since the small sample size might account for the variation and lack of statistical significance.

Re-isolation of fungal isolates revealed the isolation of *R. microsporus* as well as three fungal isolates with a close relatedness to *R. stolonifer.* Although the origin of the *R. stolonifer* isolates is unknown, they were likely environmental contaminants, as literature supports *R. stolonifer* as a causative agent in post-harvest tomato infections (1, 4, 15, 64). Re-isolation of *R. microsporus* and confirming the presence of their endosymbiotic bacteria *M. endofungorum* provides insight into the biocontrol mechanism employed by *B. velezensis.* During the *in vivo* study, we also assume that the fungal pathogen and its endosymbiotic bacteria were exposed to various secondary metabolites, including the VOCs produced by the *B. velezensis* strains. Studies have shown that the mechanisms of anti-bacterial activity of *Bacillus* VOCs include induced systemic resistance in the host plant, modulation in the pathogen’s gene expression, and changing the structure and function of the pathogen on a cellular level (44). However, the biocontrol strains seemingly did not affect the endosymbiotic bacteria; this could be due to *Bacillus velezensis* producing AMPs that mostly target gram-positive bacteria (25), and *M. endofungorum* is a gram-negative bacterium (9). Furthermore, the biocontrol mechanism of systemic resistance is most effective once the bacteria are established inside a host plant. The endosymbiotic bacteria are also potentially protected by the fungal cell wall, and in order for *B. velezensis* to target the endobacteria, it has to reach them first. *Rhizopus* spp. are known to have a chitin-rich cell wall (65), protecting their intracellular content, including the endobacteria potentially harbored in the fungal mycelium (37). Although the *B. velezensis* strains used in this study are predicted to contain biosynthetic gene clusters for the production of secondary metabolites, such as macrolactin, difficidin, and fengycin, these metabolites are only capable of changing the permeability of the cell membrane (25, 40). A chitinase enzyme could potentially aid in breaking down the glycosidic bonds in the main cell wall components of the *R. microsporus* cell wall in order for the biocontrol agent to target the endobacteria. It is also important to note that the *in vivo* biocontrol effects are more surface-level interactions than internal systematic mechanisms. Our study only focused on nature-sourced *R. microsporus* strains associated with *Mycetohabitans* endosymbionts, due to the known role of these endosymbionts in facilitating host virulence, specifically in field crops (9). However, an important future direction would be to extend research into the biocontrol efficacy of *B.* velezensis against non-host *Rhizopus* strains. To fully understand the extent of the biocontrol mechanisms employed by *B. velezensis,* it is crucial to consider the potential presence of endosymbionts in *Rhizopus* strains.

The results from the *in vivo* experiments are supported by the *in vitro* culturing experiments, as well as the VOC analysis and the whole genome annotations of the two *B. velezensis* strains. These results indicate that *B. velezensis* has the potential to inhibit the growth of *R. microsporus.* However, the inhibition was highly strain-specific. To better understand the context of our findings, it is important to consider the nutritional profile of tomatoes at various ripening stages. A recent study by Ramesh *et al*. (66) showed that as tomatoes ripen, they experience a significant increase in nutrient content. This observation supports our findings, as we inoculated our fruits at a ripe stage; this heightened period of nutrient availability could potentially facilitate the infection by *R. microsporus.* Furthermore, research by Petrasch *et al.* (4) has demonstrated that unripe tomatoes are less susceptible to pathogen infection, further supporting the idea that ripe tomatoes provide a more favorable environment for fungal pathogens. The results of the puncture inoculation reflect a *R. microsporus* infection at the inoculation site, which suggests that although a biocontrol agent was present, the pathogen could rely on the high nutrients supplied by the tomato to colonize. However, the exocarp peel inoculation revealed colonization of fungi at the tomato stem scar. A study investigating the complex structures of the stem scar found that if infected water comes into contact with the stem scar for prolonged periods, particulate matter is able to penetrate the tomato (67). The results of the exocarp peel inoculation suggest that the period when the tomatoes were left to air-dry after being submerged in bacterial suspension did not allow enough time for the bacteria to accurately protect this complex, nutrient-rich area. Little information is available on the exact composition of the stem scar, but Bartz *et al.* have proved it to be a common entry point for infection of fruit. Looking at our results for the exocarp peel inoculation, we noticed that administering the biocontrol agent, after the wounds were inflicted, was successful in redirecting the fungal infection. However, if the biocontrol agent is administered at an earlier stage, before the fruit has ripened, the biocontrol efficacy could potentially be higher. Furthermore, seeing that the surface level application of *B. velezensis* was effective in re-directing the infection in some cases further supports the idea that a deeper, systematic protection would be more robust at combating fungal infections.

### Conclusion

Our study explored the potential of *B. velezensis* strains as biocontrol agents against *R. microsporus*, a fungus responsible for post-harvest losses in tomatoes. The findings strongly emphasize the strain-specific nature of biocontrol activity. Although both *B. velezensis* strains (KV10 and KV15) demonstrated the capacity to inhibit *R. microsporus* growth *in vitro*, the efficacy varied considerably depending on the specific fungal strain encountered.

The *in vitro* experiments revealed that both direct interactions between bacteria and fungi, as well as the antifungal VOCs produced by the bacteria, played a role in inhibiting *R. microsporus*. However, the specific VOC profiles and their effectiveness differed between the two *B. velezensis* strains. This variability highlights the complex nature of the interactions between biocontrol agents and their target pathogens, suggesting that a “one-size-fits-all” approach may not be effective.

The *in vivo* experiments using tomato fruits provided further evidence of the strain-specific nature of the proposed biocontrol agents. Although *B. velezensis* showed some promise in reducing spoilage, the results were not uniform across different *R. microsporus* strains. The observation that *B. velezensis* influenced the site of infection on the tomatoes, directing fungal colonization toward the stem scar, raises interesting questions about the mechanisms of biocontrol. This finding suggests that the bacteria might be more effective in protecting certain areas of the fruit than others.

Given the complexity and strain specificity observed in this study, future research should focus on several key areas, including a comprehensive screening of a wider range of *B. velezensis* strains to identify those with the broadest and most consistent efficacy against diverse *R. microsporus* strains. Future investigation can focus on the genetic basis of antifungal activity in *B. velezensis*. This could involve analyzing the genes responsible for the production of antifungal compounds and identifying potential resistance mechanisms in *R. microsporus*. Similar efforts should be employed to address the role of the endosymbiotic bacteria harbored in the hyphae of *R. microsporus* to determine the mechanisms of inhibition needed to target the endosymbiont and whether endobacteria-free strains could also be controlled. Further efforts should also focus on optimizing the application methods of *B. velezensis*. Factors such as timing of application, concentration of bacterial inoculum, and the specific parts of the tomato fruit targeted for protection should be explored to enhance biocontrol efficacy. By addressing these research priorities, we can move toward developing more effective and reliable strategies for utilizing *B. velezensis* as a sustainable and environmentally friendly alternative to chemical fungicides for the control of post-harvest infections in tomatoes.

## Data Availability

The original contributions presented in the study are publicly available. This data can be found here: https://www.ncbi.nlm.nih.gov/genbank, accession numbers PP380448 (W2-50), PP380449 (W2-51) and PP380454 (W2-58) for *R. microsporus* strains and PP958808 (symbiont W2-50), PP958809 (symbiont W2-51) and PP958814 (symbiont W2-58) for associated endobacteria.

## SUPPLEMENTAL MATERIAL

Supplemental tables and figures spectrum.01064-25-s0001.docx Tables S1 and S2, and Figures S1 to S4.

ASM does not own the copyrights to Supplemental Material that may be linked to, or accessed through, an article. The authors have granted ASM a non-exclusive, world-wide license to publish the Supplemental Material files. Please contact the corresponding author directly for reuse.
