# Supplementary material for: The biocontrol potential of endophyte Bacillus velezensis to reduce post-harvest tomato infection caused by Rhizopus microsporus
Source: Microbiol Spectr. 2025 Oct 27;13(12):e01064-25. doi: 10.1128/spectrum.01064-25 (PMC12671129; doi:10.1128/spectrum.01064-25)
Supplement: Supplemental tables and figures — Tables S1 and S2, and Figures S1 to S4. [file spectrum.01064-25-s0001.docx]

**Supplementary material**

| 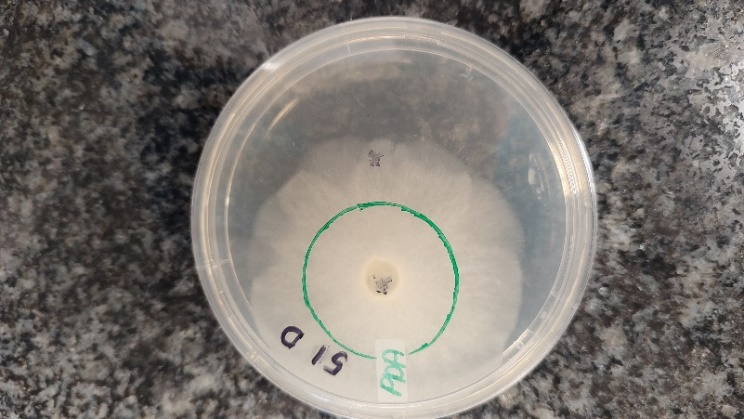**A.** *R. microsporus* (W2-51) Control | 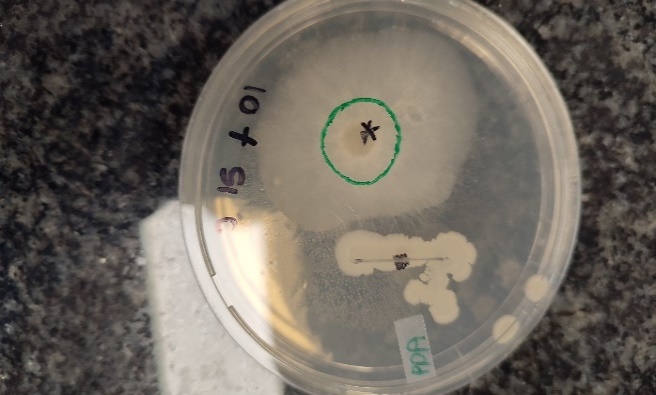**B.** *R. microsporus* (W2-51) with *Bacillus velezensis* KV10 | 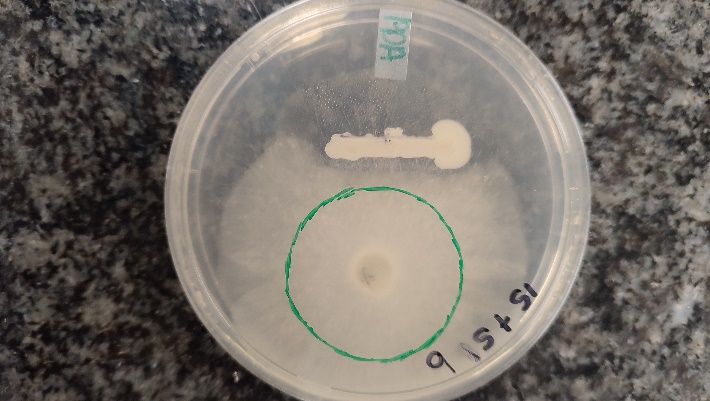  **C.** *R. microsporus* (W2-51) with *Bacillus velezensis* KV15 |
| --- | --- | --- |
| 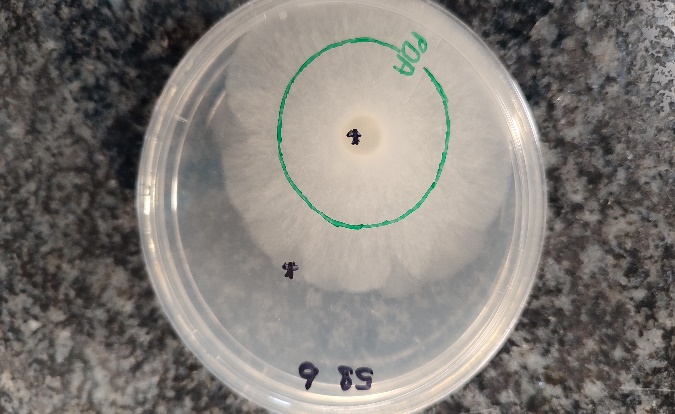**D.** *R. microsporus* (W2- 58) Control | 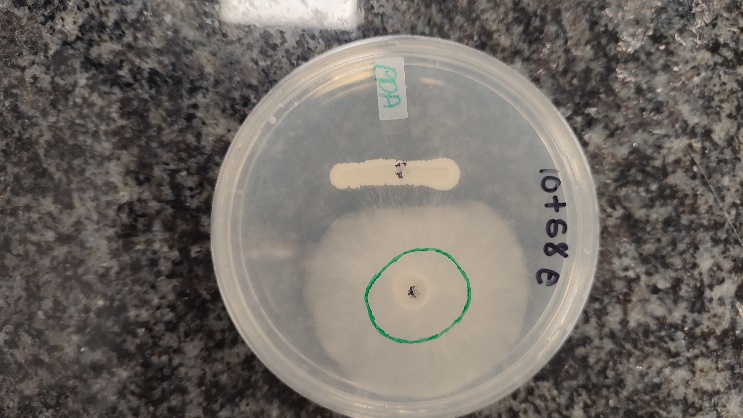**E.** *R. microsporus* (W2-58) with *Bacillus velezensis* KV10 | 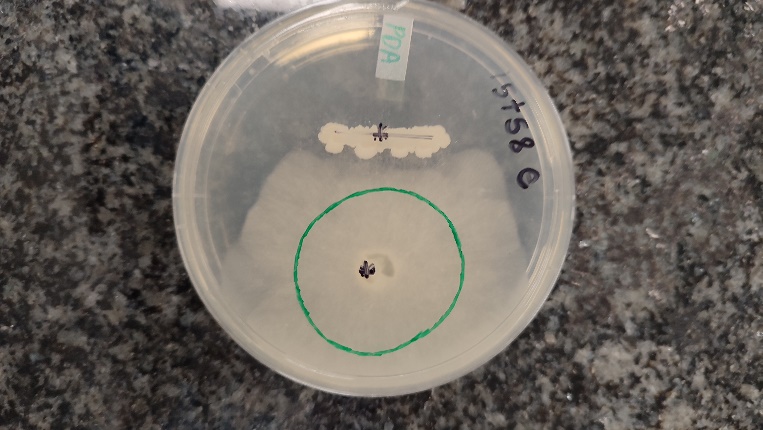  **F.** *R. microsporus* (W2-58) with *Bacillus velezensis* KV15 |
| **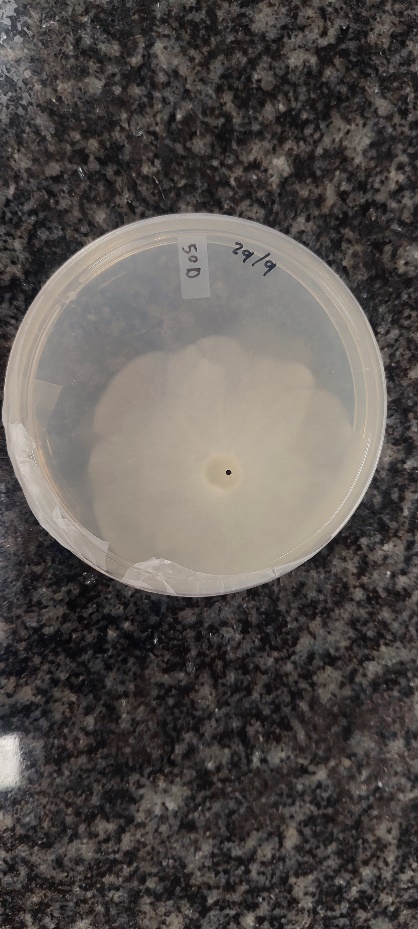**  **G.** *R. microsporus* (W2-50) Control | **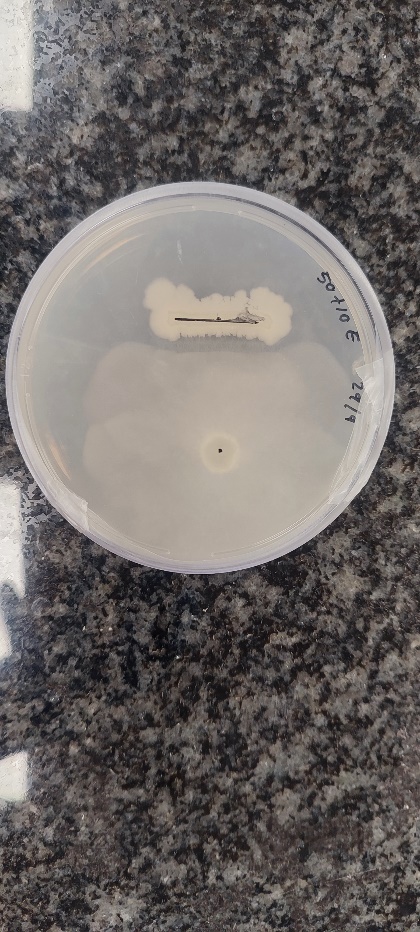**  **H.** *R. microsporus* (W2- 50) with *Bacillus velezensis* KV10 | **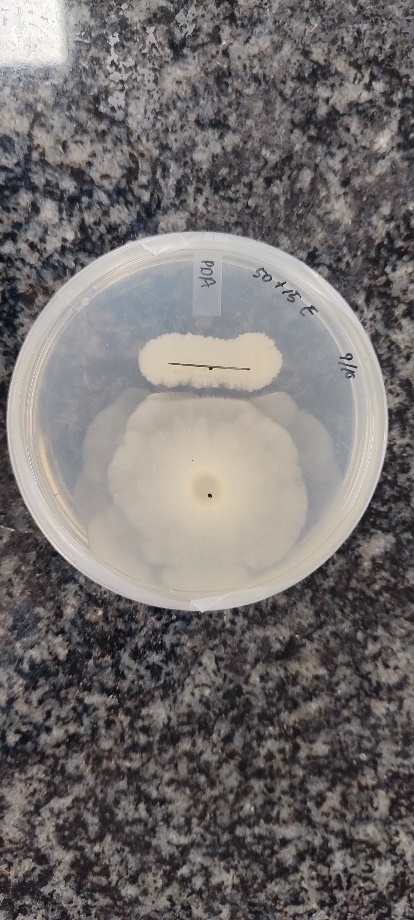**  **I.** *R. microsporus* (W2-50) with *Bacillus velezensis* KV15 |

S.Figure 1: Inhibition of radial growth after 48h incubation for three R. microsporus strains against two Bacillus velezensis strains KV10 and KV15 respectively.

| *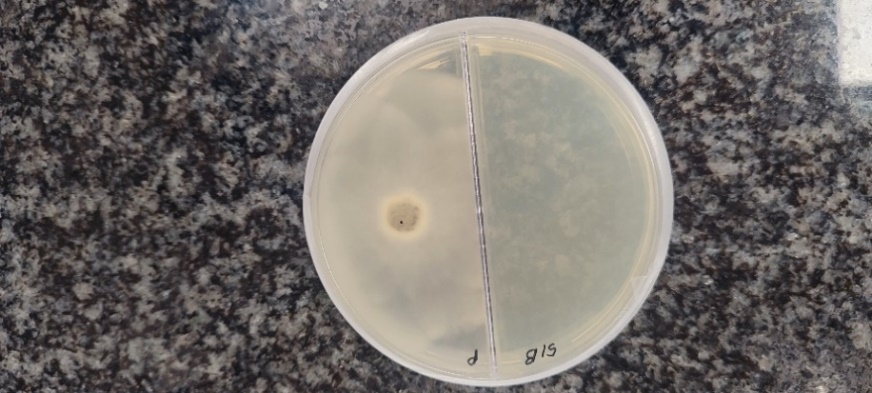*  **A.** *R. microsporus* (W2-51) Control | *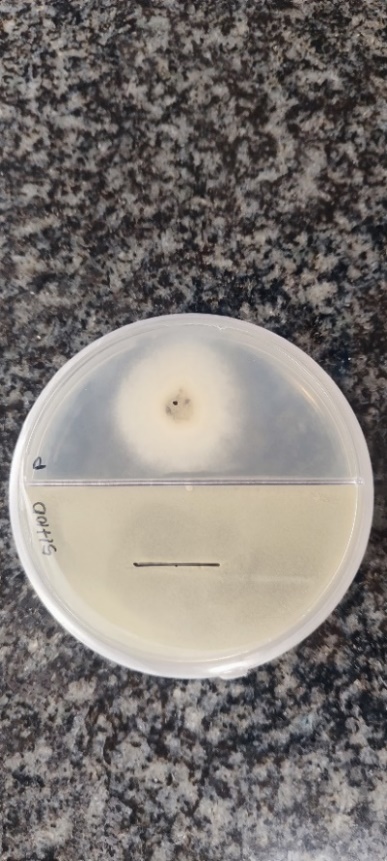***B.** *R. microsporus* (W2-51) with *Bacillus velezensis* KV10 | *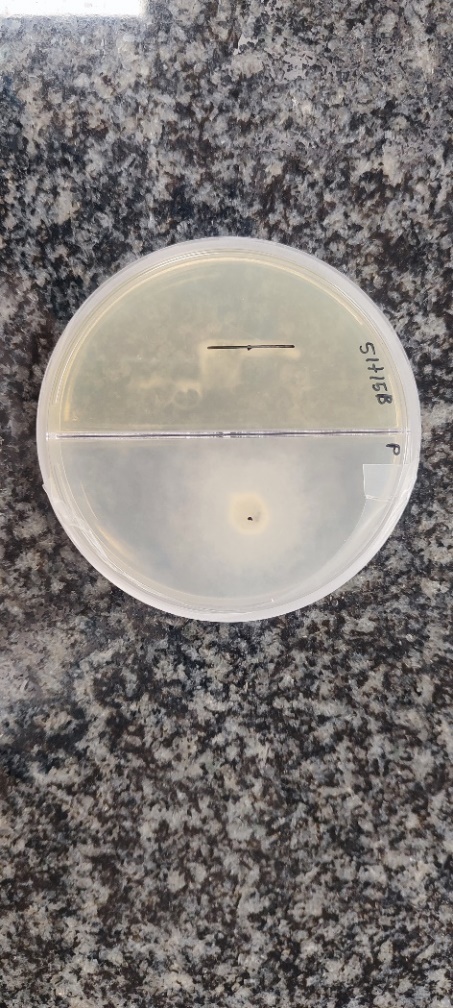*  **C.** *R. microsporus* (W2-51) with *Bacillus velezensis* KV15 |
| --- | --- | --- |
| 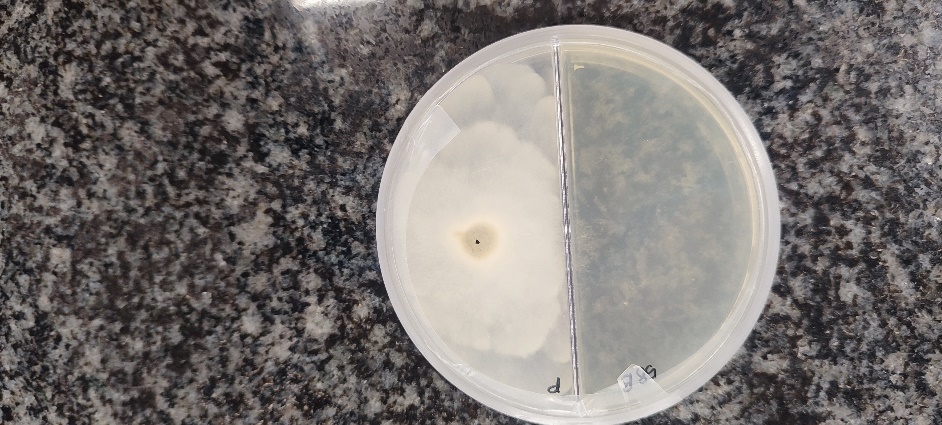**D.** *R. microsporus* (W2- 58) Control | 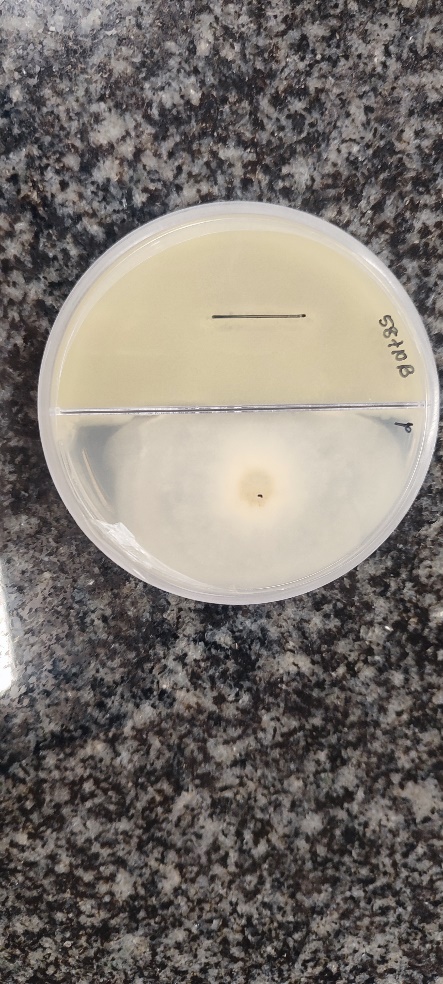**E.** *R. microsporus* (W2-58) with *Bacillus velezensis* KV10 | 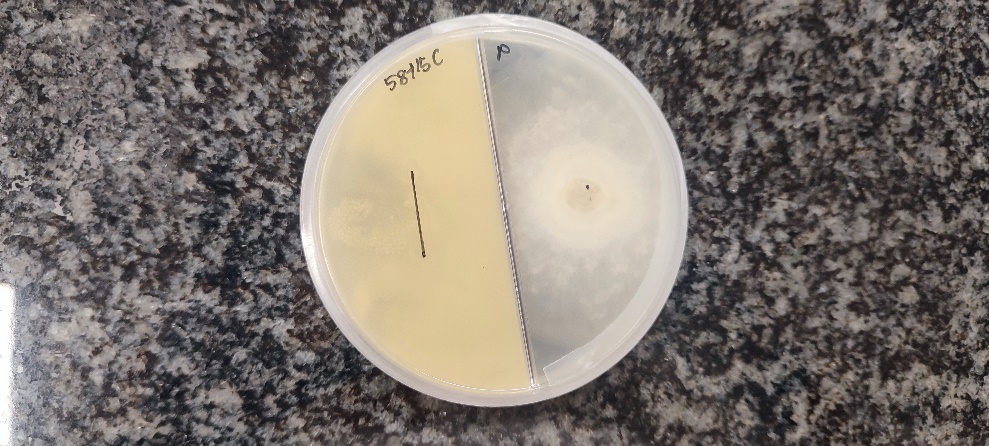  **F.** *R. microsporus* (W2-58) with *Bacillus velezensis* KV15 |
| 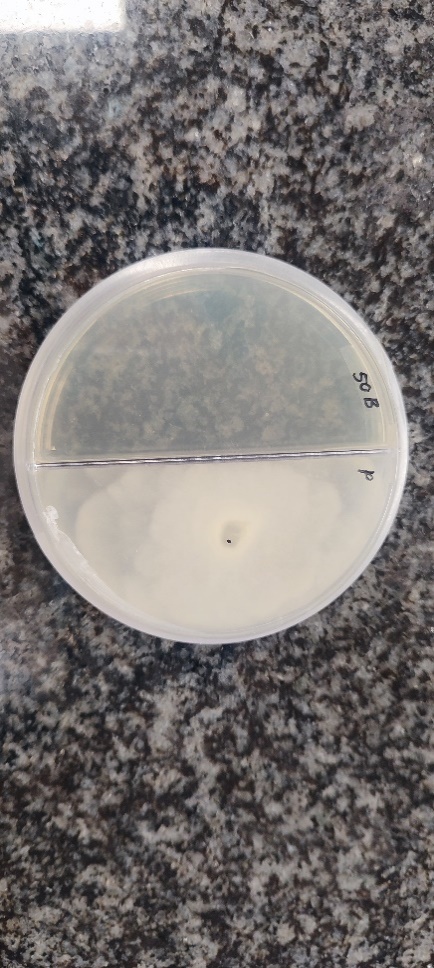**G.** *R. microsporus* (W2-50) Control | 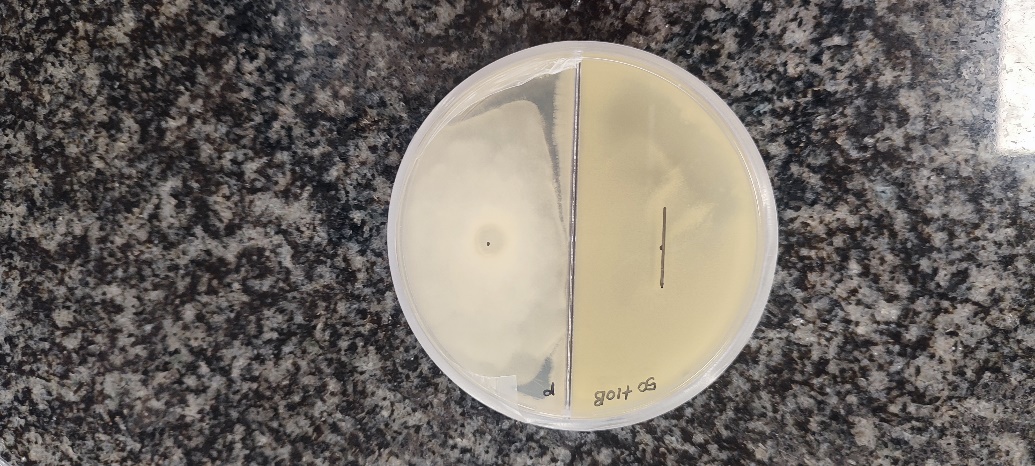**H.** *R. microsporus* (W2- 50) with *Bacillus velezensis* KV10 | 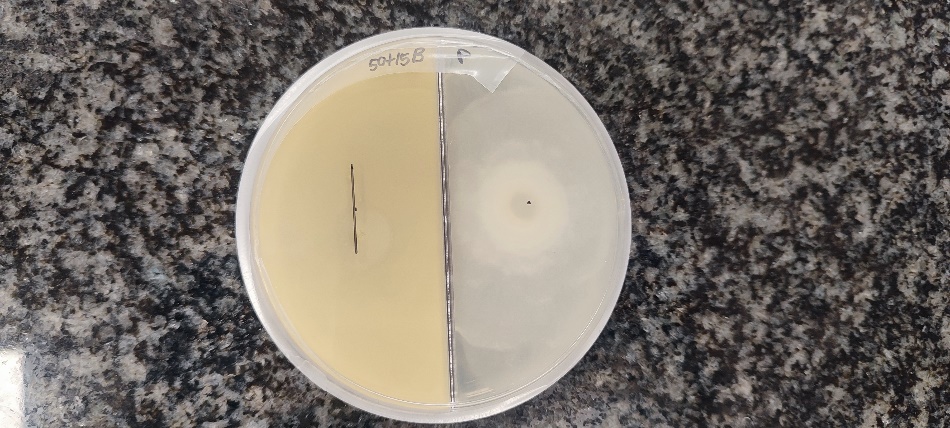  **I.** *R. microsporus* (W2-50) with *Bacillus velezensis* KV15 |

S.Figure 2: Inhibition of radial growth after 48h incubation for three R. microsporus strains against the VOCs of two B. velezensis strains KV10 and KV15 respectively.


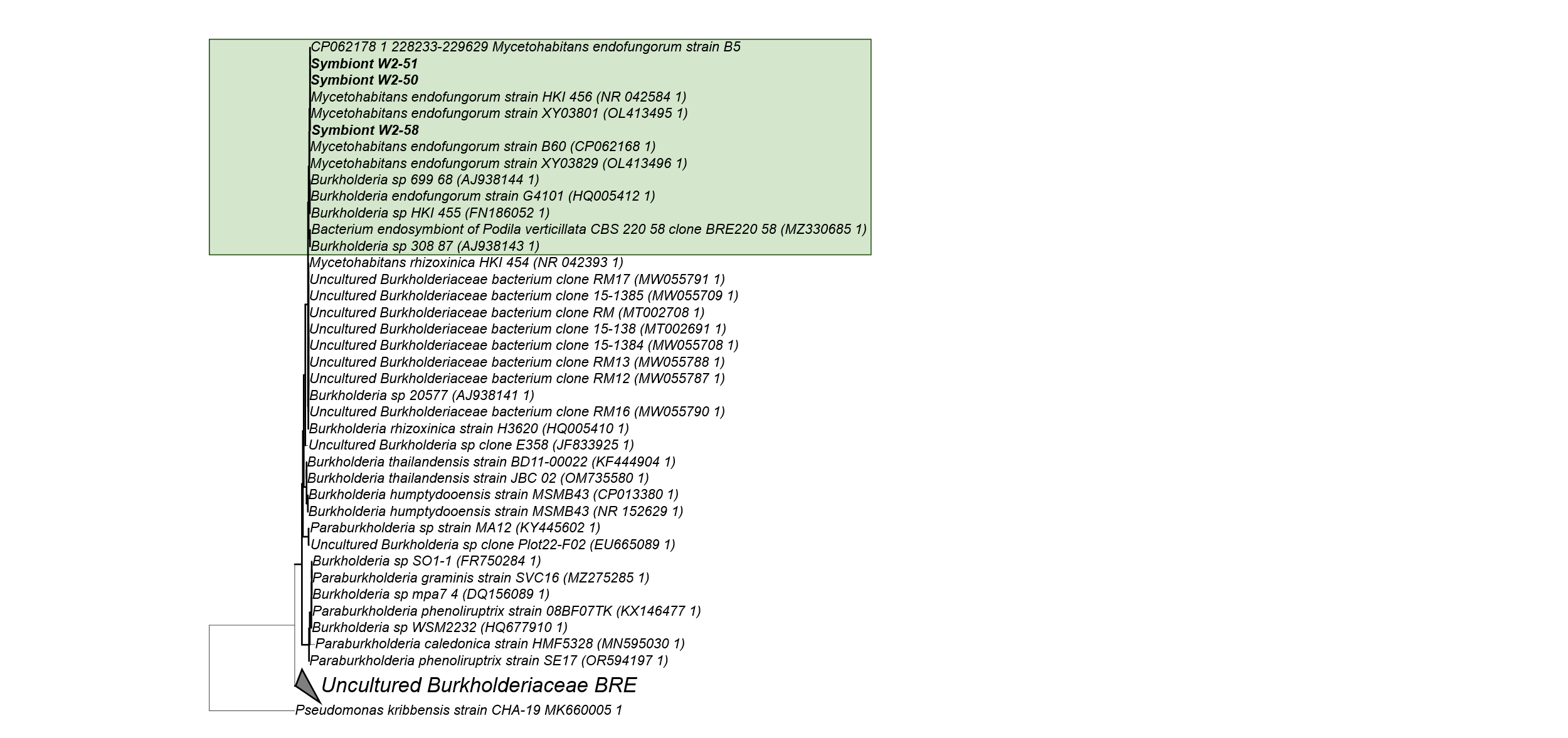


S. Figure 3: Phylogenetic tree based on the 16S rRNA gene sequence data from selected Rhizopus microsporus isolates using RAxML-GUI and applying the GTR Gamma substitution model with 1000 bootstraps. Bold branches represent bootstrap values >70%. Pseudomonas kribbensis strain CHA-19 was selected as outgroup for the phylogenetic inference.

S.Table 1: Incidence (%) of fungal growth observed in tomatoes inoculated with R. microsporus strains after they were treated with B. velezensis strains.

| **Treatment** | **Number of infected tomatoes (n=5)** | **Incidence (%)** | **Re-isolation of *Rhizopus* spp.** |
| --- | --- | --- | --- |
| Wounded uninoculated control | 3/5 | 60% | None |
| Inoculated with KV10 | 3/5 | 60% | None |
| Inoculated with KV15 | 0/5 | No spoilage | None |
| Inoculated with W2-50 | 2/5 | 40% | *R. microsporus* & *R. stolonifer* |
| Inoculated with W2-51 | 1/5 | 20% | None |
| Inoculated with W2-58 | 1/5 | 20% | None |
| Inoculated with W2-50 and KV10 | 5/5 | 100% | *R. microsporus* |
| Inoculated with W2-50 and KV15 | 1/5 | 20% | *R. microsporus* |
| Inoculated with W2-51 and KV10 | 1/5 | 20% | None |
| Inoculated with W2-51 and KV15 | 2/5 | 40% | None |
| Inoculated with W2-58 and KV10 | 1/5 | 20% | None |
| Inoculated with W2-58 and KV15 | 0/5 | No spoilage | None |

S.Table 2: Incidence (%) of fungal growth observed in tomatoes inoculated with R. microsporus W2-50 after they were treated with B. velezensis strains.

| **Treatment** | **Number of infected tomatoes (n=5)** | **Incidence (%)** | **Re-isolation of *Rhizopus* spp.** |
| --- | --- | --- | --- |
| Wounded uninoculated control | 3/5 | 60% | None |
| Inoculated with KV10 | 4/5 | 80% | None |
| Inoculated with KV15 | 3/5 | 60% | *R. stolonifer* |
| Inoculated with W1-50 | 4/5 | 80% | *R. microsporus* &  *R. stolonifer* |
| Inoculated with W1-50 and KV10 | 3/5 | 60% | *R. microsporus* |
| Inoculated with W1-50 and KV15 | 3/5 | 60% | *R. microsporus* |


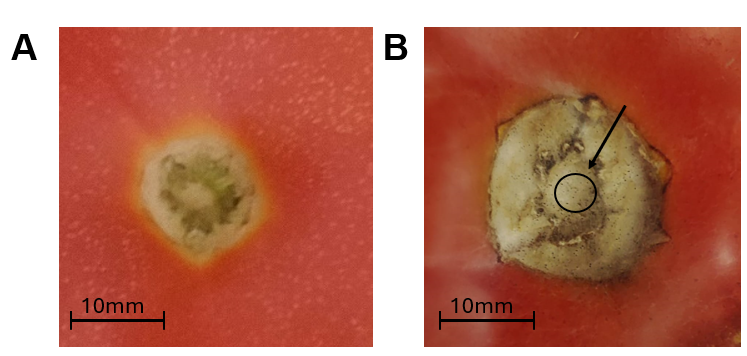


S.Figure 4: A) Image showing a healthy stem with no fungal establishment. B) Image indicating the establishment of R. microsporus W1-50 on the stem-scar of the treated tomato fruit. Visible sporangium can be seen on the surface of the stem-scar.
